# COVID-19 in School Teachers: Job Satisfaction and Burnout through the Job Demands Control Model

**DOI:** 10.3390/bs13010076

**Published:** 2023-01-16

**Authors:** Mariacarla Martí-González, María Lourdes Alcalá-Ibañez, Jose Luis Castán-Esteban, Laura Martín-Bielsa, Laura O. Gallardo

**Affiliations:** 1 Department of Psychology and Sociology, Universidad de Zaragoza, 44003 Teruel, Spain; 2 Department of Education, Universidad de Cantabria, 39005 Santander, Spain; 3 Department of Education, Universidad de Zaragoza, 44003 Teruel, Spain; 4 Colegio Profesional de Logopedas de Aragón, 50003 Zaragoza, Spain

**Keywords:** burnout, job satisfaction, school teachers, COVID-19

## Abstract

The recent pandemic has influenced teachers’ mental health and well-being. The present work follows the Job-Demands-Control model, analyzing changes in the demands, control, and social support during COVID-19, and how they influence job satisfaction, emotional exhaustion, depersonalization, and personal accomplishment among school teachers. The sample comprised 172 school teachers. The instruments applied were The Maslach Burnout Inventory (MBI), The Job Satisfaction Teacher Index (Organization for Economic Cooperation and Development (OECD), 2014), and The Demand Control Support Questionnaire (DCSQ). Job satisfaction was predicted exclusively by social support, revealing the importance of social interactions at work. Emotional exhaustion and depersonalization were predicted by job demands, showing that an increase in job demands (i.e., COVID) affects individuals’ feelings of burnout. The results show that it would be interesting to design strategies that guarantee job control in the teaching context. This would open multiple pathways to implement healthier methodological processes for teachers and the consequent research to support these processes.

## 1. Introduction

The teacher’s professional role is one of the backbones of the tasks carried out in the educational field, which has been dramatically affected by the COVID-19 pandemic. The wear-and-tear involved in this profession has already become evident in studies prior to 2020 [1,2], although it was still a little studied phenomenon. In fact, most of the scientific literature on the subject has focused on physical variables, e.g., occupational risk factors such as voice disorders [3,4] and musculoskeletal disorders/diseases [5]. However, some studies have analyzed, for example, the stress in teachers from different levels of education [2,6]. Although marked by specificities according to historical moments, different national realities, and social and cultural differences, common denominators can be identified. Some of these features include: an assigned-assumed role characterized by omnipotence (being a teacher, a parent, a counselor, someone who can “straighten crooked trees”, attend to everyone, always being available and in good spirits, smiling on the surface, affable and understanding, etc.). This description reveals the conception of the educational task. Still, it blurs its scientific profile, assuming an idealized performance decontextualized from its social requirements and, therefore, incapable of analyzing its contradictions. Accordingly, the teachers’ performance in their role leads to a level of exhaustion that affects their health and the possibility of creative responses. The above reveals the teacher’s highly demanding professional role, with low psychological and emotional compensation, implying high costs in health and well-being. There is growing interest in the negative influence of higher education institutions’ environments on university professors’ mental health due to the increasingly high expectations of their role performance (teaching, pressure to generate research, tutorials, etc.) [7].

The characteristics and performance of the teacher’s professional role have been empirically addressed through the variable of teacher leadership [8,9,10].

The COVID-19 pandemic has highlighted this issue. An important part of the studies on mental health and well-being in the school environment and the educational context has focused on the analysis of student problems and the experiences and needs of teachers [11]. In terms of job performance, the demands on teachers have increased in a very short time, and teachers have had to face a new set of situations with very little control over the evaluation of the solutions developed to deal with these demands [12].

Regardless of the concrete realities and the specificities of each country/region (infrastructures, teacher training in technology, socio-economic and psychosocial conditions, etc.), the new reality of completely virtual classes has affected teachers’ mental health [13]. One of the most influential factors was that the change occurred very rapidly, without adequate training in the use of digital resources and, in most cases, without providing proper equipment for distance classes. Such conditions are not habitual in the different online teaching modalities, which usually follow a methodically organized planning and a rigorous design and are carried out by specialists in this teaching–learning modality [14]. Another relevant element is the lack of control over the strategies implemented, which, in some cases, had not been agreed upon and were based on the teachers’ initiative/capacity/creativity.

Regardless of the teaching level, analyzing and understanding aspects of teachers’ mental health and well-being are essential for several reasons [12]: their role as the backbone of teaching–learning processes, the teaching quality, their influence on the construction of knowledge in the classroom, and also the promotion and development of student values and, of course, their own health and well-being as human beings.

Several studies have addressed the influence and consequences of the conditions derived from the COVID-19 pandemic on teachers’ mental health and/or well-being [12,15,16,17]. The present work follows the Job-Demands-Control (Support (JDC)) model [18,19] to analyze changes in the demands, control, and social support in a sample of school teachers from various Spanish schools. We will also analyze the influence of these variables on job satisfaction, emotional exhaustion, depersonalization, and personal accomplishment among school teachers.

### The Job Demands-Control Model

**Figure 1:**
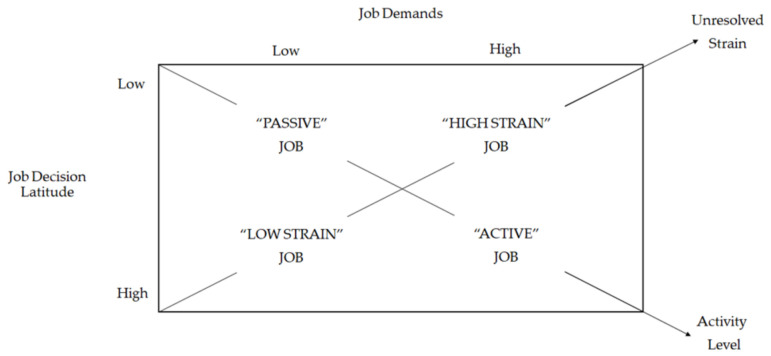
Job Demands-Control Model [18] (p. 288).

Since its creation, the JDC model [18] has generated numerous studies involving employees from diverse occupational groups and examining various outcomes This model presents a fundamental premise, which is that the employee outcomes are the result of two job characteristics: the level of job demands and the employee’s amount of job control (Figure 1) [6].

Job demands have been operationalized mainly in terms of time pressure and conflict demands and refer primarily to the workload [20]. Job control, also called decision latitude, refers to an employee’s opportunities to control their work activities. Two elements make up decision latitude: the breadth of skills used by the employee (skill discretion) and the employee’s authority to make decisions on the job (decision authority). Both elements enable the employee to influence their job and have been shown to coexist across jobs. They are, therefore, often combined into an overall measure of job control [21,22,23].

In Figure 1, the two central assumptions of the process are shown. The first process (diagonal A) constitutes the influencing element in the (poor) health of an employee, whereas the second process (diagonal B) influences the employee’s work motivation and learning behavior. Thus, four different types of work can be described in terms of job demands and job control: passive work, which combines low demands with low control; active work, which combines high demands with high control; low tension, combining low demands with high control; and high tension work, combining high demands with low control. This combination of demands and control predicts—on the one hand—the employee’s physical and mental health, with the greatest risks of health hazards in high-voltage work, and—on the other hand—the extent to which a job fosters learning and the motivation to develop new behaviors. From the above, it follows that the most positive results are expected from active work, which is considered the most desirable type, as it promotes learning and stimulates motivation [6]. However, in terms of high/low, these measures are on a continuum because they are not dichotomous; instead, they are much more complex. According to the model’s iso-strain hypothesis, a combination of high job demands, low job control, and low support as an additive effect are the variables that affect the employee’s well-being. In turn, the model’s buffer hypothesis predicts that the negative impact of high job demands on an employee’s well-being (buffering/interactive effect) can be moderated by higher social support and job control [24,25].

Despite its simplicity, this model shows great versatility, which has allowed it to be applied to a variety of different contexts to explain occupational stress. It highlights the importance of high demands to encourage employees’ creativity, motivation, and learning while guaranteeing control mechanisms that highlight good work and provide pertinent feedback. This is one of the most commonly used models to study the effects of occupational stress on workers’ health and occupational and general psychological well-being [24].

The JDC model was revised towards the end of the 1980s, integrating an essential variable in job performance: social support in the workplace [19]. Social support is conceptualized as having good relationships with coworkers and the supervisor, which provides employees with a positive social climate in which they feel supported emotionally or practically.

The model proposes that high demands act as stressors for employees. Such stress is proportional to the situations requiring high demand and employees’ low control of the situation, favored by COVID-19. Teachers have had to change their routines due to the social conditions of this situation and their low control (most of the actions have been imposed upon them). Another element is that during the lockdowns during the COVID-19 pandemic, face-to-face meetings in educational centers were abolished (now held online), and the relationship with families was also carried out through virtual tools. After returning from confinement, this situation was maintained, and online meetings at the departmental level and even tutorials with families continued to be online. Likewise, tasks within the classroom became individual, forcing the teaching staff to adapt, as this situation prevented the use of the habitual materials, given the risk of contagion. The students even had to sit separated by a specific space. All the above led teachers to consider their tasks to be highly stressful. In summary, the JDC-S model explains the impact of three key job dimensions: job demands (e.g., conflicting roles, the complexity of working tasks, time pressure), job control (e.g., decision authority, skill utilization), and social support from coworkers and supervisors.

It was also interesting to analyze the impact of these elements on possible evolutions towards burnout. Although the term burnout does not have a unanimous definition by researchers [26], in the case of this study we start from the classic definition of Maslach and Jackson [27], who conceptualize it as a three-dimensional syndrome characterized by emotional exhaustion, depersonalization, and reduced personal accomplishment.

**Figure 2:**
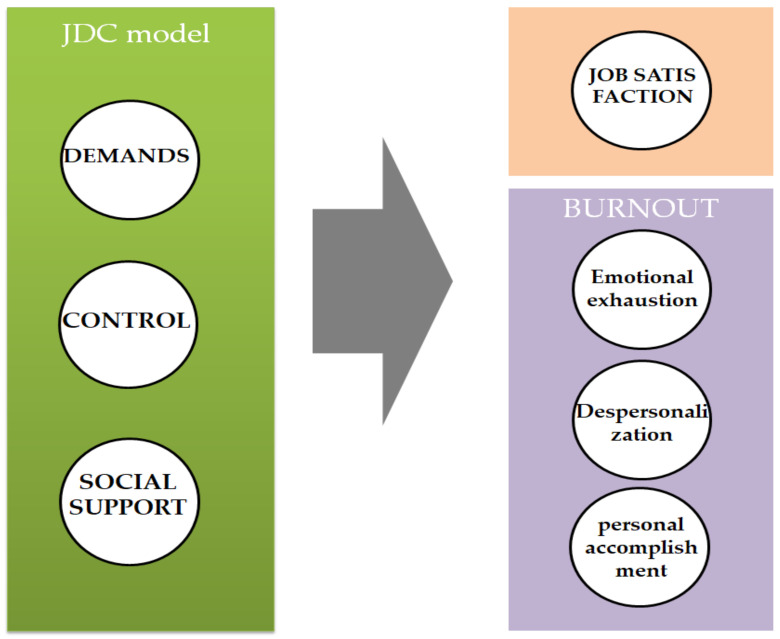
The proposed model.

Accordingly, considering that COVID-19 had modified the conditions in educational centers, the main purpose of this research was to determine whether demands, control, and social support may influence job satisfaction, emotional exhaustion, depersonalization, and personal accomplishment among school teachers (see Figure 2). Concretely, we hypothesize: (a) demands and control will positively influence burnout variables (emotional exhaustion, depersonalization, and personal accomplishment), whereas it will negatively influence job satisfaction. (b) Inversely, social support will positively influence job satisfaction and negatively influence burnout variables.

## 2. Materials and Methods

### 2.1. Participants

Participants (N = 172) were school teachers recruited in the northeast of Spain (Mage = 42.4, SD = 9.65; 74.4% females). The inclusion criterion was for the participants to have completed all the measures. The teachers belong to a total of 42 different primary and secondary schools. The participants’ mean time working in a school was 15.8 years (SD = 9.57).

All subjects gave their informed consent for inclusion before participating in the study. The study was conducted following the Declaration of Helsinki.

### 2.2. Measures

The Maslach Burnout Inventory (MBI [27]).

The MBI is a multidimensional questionnaire designed to measure burnout through three dimensions of workplace stress. The 22-item questionnaire is rated on a six-point Likert scale ranging from 0 (never) to 6 (every day). The first dimension, Emotional Exhaustion (9 items), measures feelings of being emotionally overextended and drained by one’s work. An example item is “I feel emotionally drained from my work”. The second dimension is Depersonalization. This measures an unfeeling and impersonal response toward clients (in this case, students). An example item is “I have accomplished many worthwhile things in this job”. Finally, Personal Accomplishment, the third dimension, measures feelings of competence and successful achievement in one’s work. An example item is “I don’t really care what happens to some students”. The Spanish version has been validated [25], showing good internal consistency. The Cronbach alpha of Emotional Exhaustion was 0.78, of Depersonalization was 0.76, and of Personal Accomplishment was 0.74.

The Job Satisfaction Teacher index (Organisation for Economic Cooperation and Development (OECD), 2014).

The Spanish version was used previously in the OECD Teaching and Learning International Survey (TALIS) developed in 2014 and 2018 by the OECD in several countries. The index presents 4 items rated on a four-point Likert scale ranging from 1 (totally disagree) to 4 (totally agree). Examples of items are “During this COVID-19 course, I feel satisfied with my job in this educational center” or “During this COVID-19 course, I enjoy my job”. The reliability of the index was acceptable (α = 0.75).

The Demand Control Support Questionnaire (DCSQ) [28].

The 17-item questionnaire is rated on a four-point Likert scale ranging from 1 (totally disagree) to 4 (totally agree). The DCSQ has three subscales related to the JDCS model: Psychological Demands (5 items), Control (6 items), and Social Support at work (6 items). Examples of items, respectively, are “I have to work intensely”, “My job requires ingenuity and creativity”, and “My colleagues are there for me (they support and help me)”. The Spanish version used [29] showed good internal consistency. The Cronbach alpha of Psychological Demands was 0.78, of Control was 0.78, and of Social Support at work was 0.84.

### 2.3. Procedure

Data collection was carried out during the fall of 2020. Firstly, the researchers contacted the education authority and management teams of the schools. Secondly, an informative circular, including a consent form for them to sign, was given to participants. Thirdly, the questionnaire was sent to the participants through an online link. Before completing the questionnaire, we informed the participants that all their responses were confidential and that they could leave the investigation at any time.

### 2.4. Statistical Analysis

Data were analyzed with Mplus, Version 7.11, to implement a structural equation model (SEM). Parcels were established to reduce sampling error by reducing the specific variance of each item. The parcels were configured following the recommendations of Little, Rhemtulla, Gibson, and Schoemann [30], assigning the items randomly to the parcels and then averaging them. The model is composed of 7 latent variables, with two parcels in each one. In the model, the residuals of the corresponding indicators were allowed to correlate across groups, and the first factor loading per latent variable was set to the unity to establish the scale of latent variables, as recommended by Little, Preacher, Selig, and Card [31]. Furthermore, “A value of ΔCFI smaller than or equal to 0.01 indicates that the null hypothesis of invariance should not be rejected” [32].

The SEM was analyzed in the following sequence. The model assessed whether the job demands–resources model (demands, control, and social support) influences individuals’ satisfaction and burnout (emotional exhaustion, depersonalization, and personal accomplishment). Although a multigroup SEM model does not demonstrate causality [33], this approach allows exploring and testing key issues in the pattern of relations among groups.

Considering the possible multivariate non-normality of the measures, the robust maximum likelihood (MLR) estimator was selected for model estimations [34]. Goodness of fit was tested with common fit indexes. Thus, a model fit is considered adequate when the comparative fit index (CFI) and the Tucker–Lewis index (TLI) have values of >0.90, the value of the root mean square error of approximation (RMSEA) is <0.06, and the value of the standardized root mean square residual (SRMR) is <0.08.

## 3. Results

Descriptive statistics and the correlation matrix between the model variables are shown in Table 1, which also presents reliabilities of the latent variables for the questionnaires used, with good results. It is noteworthy that sex was not related to any of the other variables, so it was not included in the model to be tested.

### Structural Equation Modeling

**Figure 3:**
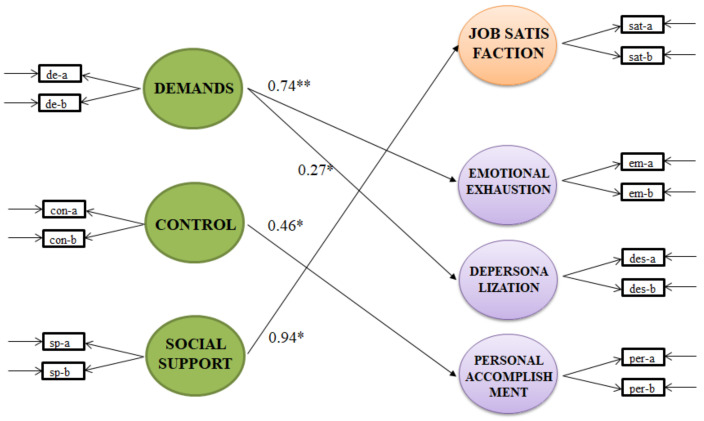
Standardized parameters of the model. Non-significant paths are omitted for presentation clarity. * *p* < 0.05, ** *p* < 0.01. de-a and de-b are the parcels that composed the latent variable of demands; con-a and con-b are the parcels that composed the latent variable of control; sp-a and sp-b are the parcels that composed the latent variable of social support; sat-a and sat-b are the parcels that composed the latent variable of job satisfaction; em-a and em-b are the parcels that composed the latent variable of emotional exhaustion; des-a and des-b are the parcels that composed the latent variable of despersonalization; and finally per-a and per-b are the parcels that composed the latent variable of personal accomplishment.

The model presented an adequate fit to the data, χ^2^(172) = 85.707, CFI = 0.965, TLI = 0.943, RMSEA = 0.056, 90% CI [0.030, 0.078], SRMR = 0.055. Figure 3 shows the standardized parameters that were significant in the model. We highlight that job satisfaction was predicted exclusively by social support, revealing the importance of social interactions at work. Emotional exhaustion and depersonalization were predicted by job demands, showing that an increase in job demands (i.e., COVID-19) affects individuals’ feelings of burnout. Personal accomplishment was predicted by control, revealing that a higher sense of control benefits personal accomplishment.

## 4. Discussion

Considering the pandemic COVID-19 situation in which the data were collected, our results showed the importance of demands, control, and social support in predicting job satisfaction and burnout among school teachers.

Looking at correlations, our results are consistent with the usual ones found in other studies in which the JDC-S model has been applied as well as the corresponding instruments [1,2,6,7,12,17,24]. Although teachers have experienced an increase in work demands derived from the pandemic and subsequent confinement, it is interesting to analyze the specifics issues of the relationship between the increase in demand and the other variables in the model. The first element is that in the case of the sample studied, the control and social support variables were positively correlated (*r* = 0.31), which could be interpreted as a positive effect of control in terms of accompaniment and human containment in the process of changes and actions devised and carried out in an improvised and forcible way. In other words, this relationship could indicate that the control was not experienced as something negative but rather as a positive and caring supervision. On the other hand, regarding the variables of emotional exhaustion, depersonalization and personal accomplishment, it is relevant to point out that our sample probably has not reached burnout. Although these are not clinical measures, high scores for emotional exhaustion and depersonalization and low scores for personal accomplishment define the syndrome. This was not the case, as the scores were medium to low. When analyzing the set of variables in the correlation matrix, the fact that the increase in demands correlated negatively (as expected) with job satisfaction and positively with depersonalization stands out. However, it is interesting to highlight that the increase in demands correlated negatively with emotional exhaustion. This fact can be due to several elements and, therefore, can be interpreted in different ways. Taking into account that while emotional exhaustion correlates negatively with demands, it also correlates positively with control, social support and job satisfaction, which raises the question of which of these variables would be predicting emotional exhaustion. On the other hand, the question arises as to whether job satisfaction and social support would be protective factors against emotional exhaustion.

Considering the model, job satisfaction was predicted exclusively by social support, revealing the importance of social interactions at work. The rapid modification of the task demands that took place, plus the high degree of unpredictability and uncertainty of the consequences of the unprecedented confinement/pandemic, increased the relevance of social support. In cases of crisis or substantial changes in the conditions of one’s role, human social support becomes highly relevant. Social support—the bond with others, realizing that others experience the same situation, recognizing common problems and anxieties—facilitates success in the face of such rapid changes. In this case, an important element of social support’s prediction of job satisfaction may involve the entailed emotional containment. Social support takes place in a context of dialogue, support and interaction between equals where feedback about the practice could emerge, which would influence job control.

These results are consistent with those found by Johnsen et al. [35] in a study that examined the relationship between directive and non-directive social support, and subjective health complaints, job satisfaction, and the perception of job demands and job control. In this sense, a distinction can be made between directive social support, where the provider assumes responsibility, and non-directive social support, where the recipient is in control [35]. A nuance is the quality and specific characteristics of social support because, in circumstances of this magnitude, how support is provided could have a more decisive influence than the amount of support received. In our case, we are referring to non-directive social support, which can be an important factor when seeking to improve the psychosocial work environment.

The influence of social support on job satisfaction has received extensive empirical support from various models [36,37,38,39,40]. The results of this study are consistent with those found in other works and reveal this variable as a moderator of job satisfaction, especially in times of crisis. Social support involves the resources provided by other people [40] and is an eminently interactive process that enhances increased self-esteem, a sense of capacity, and adequate coping strategies. Social support also constitutes a space where one can demonstrate competence or accept change physically or psychosocially [40].

Closely related to social support, as expected, emotional exhaustion and depersonalization were predicted by job demands. Undoubtedly, the increased demand for work due to lockdowns had a decisive influence on individuals’ feelings of burnout. In fact, at least three of the elements described by Karasek [18,20] that may potentially increase job demands were present: increased task complexity, more conflictive situations (teachers frequently had to deal with parents’ and students’ complaints daily, pressure from academic directors, etc.) and time pressure (i.e., the record time in which study plans designed for a face-to-face term had to be adapted to different online versions).

In this sense, although not directly evaluated, violence could also affect both the increase in task complexity and conflicts (e.g., the complexity of job demands). Berlanda et al. [41] concluded that teaching is one of the most stressful occupations, as intense psychological demands—for example, the need to develop positive relationships with students and families, manage groups, etc.—are inherent to it. Their study revealed the role of job demands in determining teachers’ experience of violence. We refer to psychological, relational, structural, or even cultural violence rather than physical violence. The social anxiety of this context, influenced by the high uncertainty, could increase the teaching staff’s experience of violence, which could change their perception of the job demands. The psychological cost of managing nonphysical violence may have played a significant role—in terms of job demands—in teachers’ depersonalization and emotional exhaustion, as it is less obvious and more difficult to identify, recognize, and manage. This element could be addressed in future research, even with the limitations of a retrospective study.

In any case, a large number of previous studies illustrate that high job demands and workloads are significant predictors of mental health and stress [41,42,43,44,45] particularly in the teaching context [41]. Therefore, the results of this study are consistent with the findings of previous research.

In such a complex situation, control was precisely the predictor of personal accomplishment. The educational contexts/schools that implemented different variations of the evaluation process, allowed supervising the strategies implemented, or carried out follow-up procedures, showed greater personal achievement.

The main limitation of this study is the type of design, since as it is a cross-sectional design, data are available from a single moment in time, and it would be interesting to evaluate data measured by these instruments over time in order to establish deeper analyzes and richer comparisons. In any case, this element can be a line of future research.

The model is traditionally used to quantify how employees perceive the risk of suffering an accident or illness as a result of their work activity. In the case of COVID-19, there are two components: on the one hand, the direct component derived from the pandemic itself and the posed health risks and, on the other hand, the stress and work overload from having to adapt tasks at work to the situation derived from the imposed social restrictions.

Thus, the situation has an objective component but also a subjective one. This exceptional situation, together with the continuous news of the media, can distort individuals’ perception of the risks to their health. Therefore, a first theoretical conclusion is the applicability of the JDC-S model to study aspects of occupational health not focused exclusively on the real presence of a problem, but rather on the perceptions of subjective risk. This approach extends the lines of research towards the analysis of the perceived potential of any health problem, raising new research coordinates that could be developed in the future.

## 5. Conclusions

We highlight the importance of social support to deal with crises in any context, particularly in the labor context. In fact, this strategy is highly applicable in the teaching context, where the teaching outcomes can have such a subjective—in many cases, nuanced— evaluative component, not only in crises such as COVID-19, but also as a part of work routines in ordinary settings. Another relevant conclusion is the considerable increase in teachers’ job demands suffered throughout their adaptation to the new conditions derived from the health crisis. These increased job demands have not been recognized in their fair measure, as in other professions such as health workers. In many cases, this situation has come to be considered normal, and, in other sadly mediatic cases, teachers’ adaptation processes have been discredited. Finally, the role of job control in personal accomplishment is worth highlighting. One of the distinctive elements of this work and that has contributed to the analysis carried out is the inclusion of the three proposed measures (The Maslach Burnout Inventory (MBI), The Job Satisfaction Teacher Index (Organization for Economic Cooperation and Development (OECD), 2014), and The Demand Control Support Questionnaire (DCSQ)). This has allowed a more holistic approach to studying the complex experiences and emotions that have emerged from the pandemic. It would be interesting to design strategies that guarantee job control in the teaching context. This would open multiple pathways for the implementation of healthier methodological processes for teachers and the consequent research to support these processes.

## Figures and Tables

**Table 1:** Means, standard deviations, and correlations between latent variables.

Variables	M	SD	α	1	2	3	4	5	6	7	8
1. Demands	14.98	2.68	0.69								
2. Control	16.01	1.92	0.56	−0.13							
3. Social Support	19.67	3.26	0.86	−0.18 *	0.31 **						
4. Job satisfaction	13.74	2.33	0.87	−0.16 *	0.26 **	0.73 **					
5. Emotional Exhaustion	20.46	11.53	0.87	−0.57 **	−0.22 **	−0.24 **	−0.25 **				
6. Depersonalization	2.81	4.04	0.60	0.18 *	−0.09	−0.11	−0.08	0.42 **			
7. Personal Accomplishment	39.96	6.19	0.80	−0.04	0.33 **	0.27 **	0.34 **	−0.05	−0.02		
8. Sex				0.03	0.03	0.06	0.08	0.13	−0.11	0.10	

* *p* < 0.05. ** *p* < 0.001.

## Data Availability

Datasets analysed or generated during the study are under the control of the investigators at the correspondence author.
